# Relationship between oligoarginine-induced membrane damage of single *Escherichia coli* cells and entry of the peptide into the cytoplasm

**DOI:** 10.1016/j.bbrep.2024.101777

**Published:** 2024-07-09

**Authors:** Sabrina Sharmin, Md. Zahidul Islam, Masahito Yamazaki

**Affiliations:** a Integrated Bioscience Section, Graduate School of Science and Technology, Shizuoka University, Shizuoka, 422-8529, Japan; b Nanomaterials Research Division, Research Institute of Electronics, Shizuoka, 422-8529, Japan; c Dept. of Biotechnology and Genetic Engineering, Jahangirnagar University, Savar, Dhaka, 1342, Bangladesh; d Dept. of Science, Graduate School of Integrated Science and Technology, Shizuoka University, Shizuoka, 422-8529, Japan

**Keywords:** Cell-penetrating peptides, Antimicrobial activity, Membrane damage, Entry of peptides, *E. coli* cells, Giant unilamellar vesicles

## Abstract

Cell-penetrating peptides (CPPs) can enter the cytosol of eukaryotic cells without killing them whereas some CPPs exhibit antimicrobial activity against bacterial cells. Here, to elucidate the mode of interaction of the CPP nona-arginine (R_9_) with bacterial cells, we investigated the interactions of lissamine rhodamine B red-labeled peptide (Rh-R_9_) with single *Escherichia coli* cells encapsulating calcein using confocal laser scanning microscopy. After Rh-R_9_ induced the leakage of a large amount of calcein, the fluorescence intensity of the cytosol due to Rh-R_9_ greatly increased, indicating that Rh-R_9_ induces cell membrane damage, thus allowing entry of a significant amount of Rh-R_9_ into the cytosol. To determine if the lipid bilayer region of the membrane is the main target of Rh-R_9_, we then investigated the interaction of Rh-R_9_ with single giant unilamellar vesicles (GUVs) comprising an *E. coli* polar lipid extract containing small GUVs and AlexaFluor 647 hydrazide (AF647) in the lumen. Rh-R_9_ entered the GUV lumen without inducing AF647 leakage, but leakage eventually did occur, indicating that GUV membrane damage was induced after the entry of Rh-R_9_ into the GUV lumen. The Rh-R_9_ peptide concentration dependence of the fraction of entry of Rh-R_9_ after a specific interaction time was similar to that of the fraction of leaking GUVs. These results indicate that Rh-R_9_ can damage the lipid bilayer region of a cell membrane, which may be related to its antimicrobial activity.

## Highlights

• Fluorescent probe-labeled R_9_ (Rh-R_9_) induces cell membrane damage of *E. coli* cells.
• Most Rh-R_9_ entered the cytoplasm of *E. coli* cells through the cell membrane damage.
• Rh-R_9_ entered the lumen of giant unilamellar vesicles (GUVs) without membrane damage.
• After the entry of Rh-R_9_ into GUV lumen membrane damage occurred in the GUV membrane.
• Rate of Rh-R_9_-induced membrane damage increased with increasing its concentration.

## 1 Introduction

Cell-penetrating peptides (CPPs) can enter the cytosol of eukaryotic cells without killing the cells [[1], [2], [3], [4]]. Two types of CPPs have been characterized: amphipathic CPPs such as penetratin and transportan 10 (TP10) [[5], [6], [7]], and arginine-rich CPPs such as TAT peptides and oligoarginine (R_n_) [[8], [9], [10]]. CPPs might enter cells via endocytosis (such as receptor-mediated endocytosis and macropinocytosis), or by direct translocation across the cell membrane [[1], [2], [3], [4],^11^,^12^]. Even in the former case, CPPs must translocate across the lipid bilayer regions of the parts of cell membranes such as endosomes. Several mechanisms of the translocation of CPPs across the lipid bilayer regions of these membranes have been proposed [[1], [2], [3], [4]].

CPPs exhibit antimicrobial activity against bacterial cells [4,13]. For example, TP10 has antimicrobial activity against *Staphylococcus aureus*. During this interaction, the membrane-impermeable fluorescent probe SYTOX green enters the cytoplasm, indicating that TP10 damages the cell membrane [13]. This action of CPPs appears similar to that of antimicrobial peptides (AMPs). The interaction of fluorescent probe-labeled TP10 with *S. aureus* cells increases the fluorescence intensity (FI) of the cells, suggesting that TP10 enters the cytosol [13]. Other CPPs exhibit antimicrobial activities, including penetratin, pVEC [13], MAP [14], and TAT [15]. The efficiency of cell penetration of various CPPs into *Escherichia coli* cells and their cytotoxicity have been examined [16]. It is well known that after cell death cell membrane is damaged, resulting in high membrane permeation. Thus, if CPPs do not induce membrane damage immediately after their interaction with cells, the cell membrane is damaged after cell death. It is therefore difficult to conclude whether the main cause of cell death is the entry of CPPs into the cytosol or CPPs-induced direct damage of bacterial cell membrane.

Recently, we have developed a new method to simultaneously monitor the entry of peptides into the cytosol and the peptides-induced membrane damage in single bacterial cells using confocal scanning laser microscopy (CLSM) [17]. Using this method, we succeeded in simultaneous measurement of the entry of a fluorescent probe (lissamine rhodamine B red (Rh))-labeled lactoferricin B (4–9) (i.e., Rh-LfcinB (4–9)), which is a well-known AMP [18], into cytosol of single *E. coli* cells and the cell membrane damage in single cells. Rh-LfcinB (4–9) entered the cytosol without damaging the cell membrane, indicating that it is a CPP-type AMP.

Here, we applied this method to elucidate the mode of interaction of nona-arginine (R_9_) with bacterial cells. First, we investigated the interaction of fluorescent probe-labeled R_9_ (i.e., Rh-R_9_) with single *E. coli* cells encapsulating the fluorescent probe calcein using CLSM to simultaneously monitor the entry of Rh-R_9_ into the cytosol and membrane damage in single cells. Rh-R_9_ induced cell membrane damage, allowing entry of a significant amount of Rh-R_9_ into the cytosol. To determine if the lipid bilayer region of the membrane is the main target of Rh-R_9_, we then investigated the interaction of Rh-R_9_ with giant unilamellar vesicles (GUVs) comprising an *E. coli* polar lipid extract (*E. coli*-lipid) by using the single GUV method for CPPs [19,20]. The lipid composition of these GUVs is phosphatidylethanolamine (PE), phosphatidylglycerol (PG), and cardiolipin (67/23/10; weight ratio) [21], similar to that of the *E. coli* cell membrane. *E*. *coli*-lipid-GUVs have been used extensively for various experiments such as the interaction of AMPs with *E. coli* cells [17,22,23]. Based on the results, we discussed the mode of interaction of Rh-R_9_ with *E. coli* cells.

## 2 Materials and methods

### 2.1 Materials

Dioleoyl-PG (DOPG), dioleoylphosphatidylcholine (DOPC), and *E. coli* polar lipid extract were purchased from Avanti Polar Lipids, Inc. (Alabaster, AL, USA). Calcein-acetoxymethyl (calcein-AM) and AlexaFluor 647 hydrazide (AF647) were purchased from Invitrogen (Carlsbad, CA, USA). Bovine serum albumin (BSA) and Nutrient Broth Medium were purchased from Fuji Film Wako Pure Chemical Co. (Osaka, Japan). Rh succinimidylester was purchased from AAT Bioquest Inc. (Sunnyvale, CA, USA). EZ rich medium was purchased from TEKnova (Hollister, CA, USA).

The synthesis of R_9_ has been described previously [24]. Rh-R_9_, in which a Rh group was attached at the N-terminus of R_9_, was prepared by the reaction of Rh succinimidylester with R_9_-peptide resin according to the previously reported method [18]. The mass spectrum of Rh-R_9_ was obtained by liquid chromatography-mass spectrometry analysis [18], and its measured mass was 2073.2 ± 0.1 Da, consistent with its molecular mass. The concentration of Rh-R_9_ in aqueous solution was determined by absorbance measurements at 568 nm using a molar extinction coefficient of 95,000 M^−1^cm^−1^.

### 2.2 Interaction of Rh-R_9_ with single *E. coli* cells

*E. coli* (JM-109) suspensions were prepared in EZ rich medium (containing 50 mM NaCl [25]) without nucleobase (ACGU). Calcein was encapsulated in the cytosol of *E. coli* cells using calcein-AM [22,26], then the cells were transferred to a hand-made chamber with a coverslip coated with poly-l-lysine [22].

Single *E. coli* cells interacting with Rh-R_9_ in EZ rich medium were observed under a confocal laser scanning microscope (FV-1000, Olympus, Tokyo, Japan) at 25 ± 1 °C using a thermoplate stage (Tokai Hit, Shizuoka, Japan) [17]. Rh-R_9_ solution (in medium) was applied to the neighborhood of single cells continuously using a glass micropipette (20 μm diameter) [17].

### 2.3 Interaction of Rh-R_9_ with single GUVs

We applied the single GUV method for CPPs using GUVs encapsulating small GUVs [17,19,20] to make simultaneous measurement of Rh-R_9_-induced pore formation in the GUV membrane and the entry of Rh-R_9_ into the GUV lumen. *E. coli*-lipid-GUVs were prepared in buffer (10 mM HEPES, pH 7.5, 1 mM EGTA, 50 mM NaCl) containing 0.10 M sucrose, small DOPG/DOPC (2/8; molar ratio)-GUVs, and 6.0 μM AF647 using the natural swelling method. The GUVs were purified by the membrane filtering method [17,19,20].

The purified GUV suspension was transferred to a chamber with glass surfaces coated with BSA [19,20]. Single GUVs interacting with Rh-R_9_ were observed under a confocal laser scanning microscope at 25 ± 1 °C using a thermoplate stage [17] and a peptide solution (in buffer containing 0.1 M glucose) was applied to the neighborhood of single GUVs continuously through a micropipette [17]. The methods of the experiments and analysis were described in Refs. [17,19,20].

We also examined the interaction of Rh-R_9_ with *E. coli*-lipid-GUVs without containing small GUVs to measure the Rh-R_9_-induced pore formation in the GUV membrane using the same method described above. For this purpose, *E. coli*-lipid-GUVs were prepared in buffer containing 0.10 M sucrose and 6.0 μM AF647 using the natural swelling method.

## 3 Results and discussion

### 3.1 Rh-R_9_-induced membrane damage of single *E. coli* cells and entry of Rh-R_9_ into cytosol

We used a standard method [27] to measure the minimum inhibitory concentration (MIC) of Rh-R_9_ against *E. coli* cells (JM-109) in Nutrient Broth medium and obtained a value of 10 ± 2 μM, indicating that Rh-R_9_ exhibits antimicrobial activity against *E. coli* cells.

**Fig. 1:**
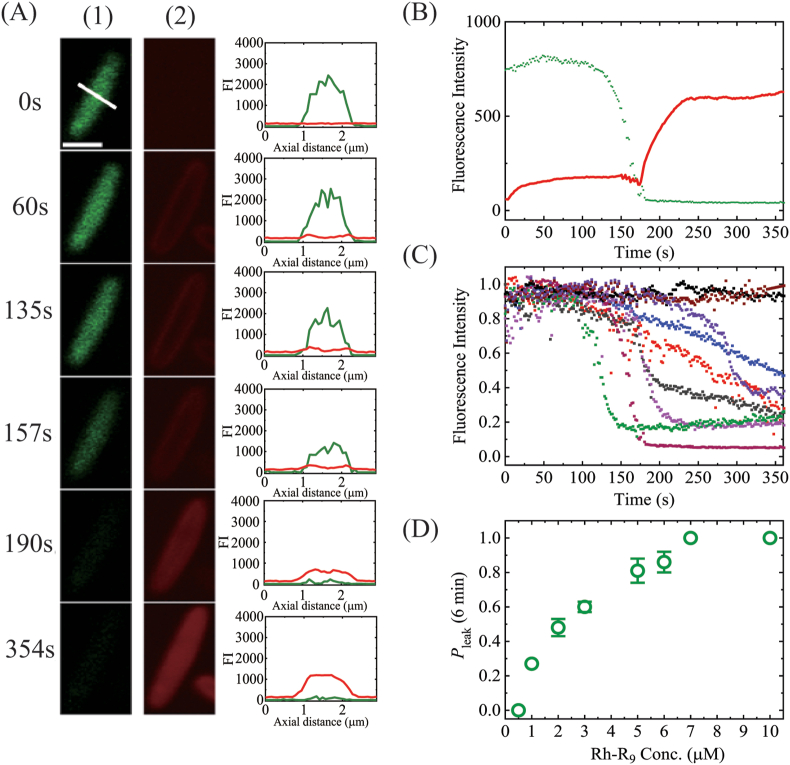
Rh-R_9_-induced membrane damage of single *E. coli* cells and the entry of Rh-R_9_ into their cytosol. (A) CLSM images of a cell due to (1) calcein, and (2) Rh-R_9_ during its interaction with 2.0 μM Rh-R_9_. The interaction time is described in the left of each image. Bar, 2 μm. For each image, the FI line profile along the white line (indicated in the 0 s image in Fig. 1A (1)) is shown in its righthand. Red line and green line correspond to the FI line profile due to Rh-R_9_ and calcein, respectively. (B) Change in the FI of the cell (shown in panel A) over time. Red line and green squares correspond to the FI of the whole cell due to Rh-R_9_ and calcein, respectively. (C) Other examples of FI due to calcein of several cells over time. (D) Rh-R_9_ concentration dependence of *P*_leak_ (6 min). Mean values and SDs are shown. (For interpretation of the references to colour in this figure legend, the reader is referred to the Web version of this article.)

To elucidate the mode of interaction of Rh-R_9_ with bacterial cells, we investigated by CLSM the interaction of Rh-R_9_ with single *E. coli* cells containing calcein in their cytoplasm at 25 °C. Fig. 1A shows the result obtained using 2.0 μM Rh-R_9_. The fluorescence intensity (FI) of a cell due to calcein initially remained almost unchanged up to 125 s, then it started to decrease gradually (Fig. 1A and B), indicating that Rh-R_9_ damages the cell membrane at 125 s and calcein then leaks from the cell. However, the FI of the whole cell (due to Rh-R_9_) increased with time and plateaued at ∼50 s, and at 175 s the FI restarted to increase up to 250 s (Fig. 1A (2), 1B). The distribution of FI due to calcein or to Rh-R_9_ in the cell was obtained by creating FI profiles along the white line (indicated in the 0 s image in Fig. 1A (1)) for each image (right figures in Fig. 1A). Between 60 and 157 s, the FI due to Rh-R_9_ had two maxima, one each at the rim of the cell moving horizontally across the cell (red lines in the FI line profile), which is supported by the images in Fig. 1A (2) where the red rims of the cell are clearly observed. The rim of the cell corresponds to the membranes, and its FI (i.e., the rim intensity) is due to the binding of Rh-R_9_ to the membranes. Initially, the rim intensity was larger than the FI of the cytosol, indicating no significant entry of Rh-R_9_ into the cytosol, whereas at 190 s and 354 s, the FI of the cytosol increased and thus the two maxima could no longer be clearly observed, indicating that Rh-R_9_ had entered the cytosol. Therefore, the initial small increase in FI of the whole cell and its rapid increase after 175 s (Fig. 1B) correspond to the binding of Rh-R_9_ to the membranes and the entry of Rh-R_9_ into the cytosol after 175 s, respectively. This method to determine the onset time of entry of peptides into cytosol has previously been used [17]. Fig. 1B shows that calcein leakage was almost complete after 180 s, indicating that the entry of a significant amount of Rh-R_9_ into the cytosol starts after leakage of a large amount of calcein. We repeated this experiment with 13 cells (*n* = 13), and found that leakage occurred in 7 cells within 6 min and so the fraction of leaking cells relative to all examined cells after 6 min interaction (*P*_leak_ (6 min)) was 0.54. Fig. 1C shows the time course of the FI due to calcein in several cells. The time of leakage onset clearly differed. In most cells, the relationship between leakage and entry was similar to that shown in Fig. 1B (i.e., after a large amount of calcein leaked, a significant amount of Rh-R_9_ entered), indicating that Rh-R_9_ induces cell membrane damage, allowing Rh-R_9_ to enter the cytoplasm. We performed two independent experiments (*N* = 2), and obtained similar results, with a mean value and standard deviation (SD) of *P*_leak_ (6 min) of 0.48 ± 0.05.

Next, we investigated the dependence of Rh-R_9_-induced calcein leakage from cells on its concentration. At 0.5 μM peptide no leakage was observed, whereas at and above 1.0 μM, *P*_leak_ (6 min) increased with peptide concentration, and at 7.0 μM *P*_leak_ (6 min) became 1.0 (Fig. 1D), indicating that the rate of Rh-R_9_-induced cell membrane damage increases with peptide concentration.

### 3.2 Rh-R_9_-induced membrane damage of *E. coli*-lipid-GUVs and entry of Rh-R_9_ into GUV lumen

We investigated the cause of Rh-R_9_-induced cell membrane damage by examining the interaction of Rh-R_9_ with *E. coli*-lipid-GUVs (i.e., mother GUVs) containing small GUVs and the water-soluble fluorescent probe AF647 in the mother GUV lumen [17,19,20]. This single GUV method enables the simultaneous measurement of the time course of the entry of Rh-R_9_ peptide into the GUV lumen (by detecting the fluorescence of small GUVs due to fluorescent probe-labeled peptides) and the time course of AF647 leakage. These measurements provide information on the relationship between the entry of peptides into a GUV lumen and peptide-induced membrane damage such as pore formation.

**Fig. 2:**
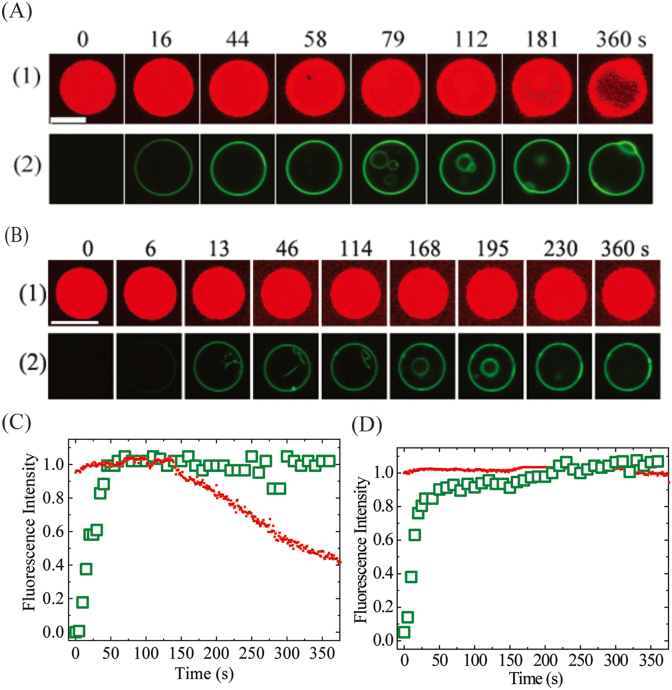
Rh-R_9_-induced membrane damage of single *E. coli*-lipid-GUVs and the entry of Rh-R_9_ into the GUV lumen. (A) (B) CLSM images of a GUV due to (1) AF647 and (2) Rh-R_9_ during its interaction with (A) 5.0 μM Rh-R_9_ and (B) 7.0 μM Rh-R_9_. The interaction time of Rh-R_9_ with the GUV is shown above each image. Bar in panel A and B, 10 μm. (C) and (D) show the change in FI of the GUV over time shown in panel A and B, respectively. Green open squares and red solid squares correspond to the FI of the GUV rim due to Rh-R_9_ and of the GUV lumen due to AF647, respectively. (For interpretation of the references to colour in this figure legend, the reader is referred to the Web version of this article.)

Fig. 2A shows the results for the interaction of 5.0 μM Rh-R_9_ with single *E. coli*-lipid GUVs in buffer. After initiating the interaction, the FI of the GUV lumen due to AF647 remained unchanged for 130 s, then gradually decreased with time (Fig. 2A (1), 2C). The FI reached 40 % of its initial intensity at 360 s, whereas the spherical shape and size of the GUV remained unchanged. This result indicates that Rh-R_9_ induces membrane damage at 130 s, allowing the gradual leakage of AF647 [19]. In contrast, the rim intensity of the GUV due to Rh-R_9_ increased rapidly and plateaued at 40 s (Fig. 2C). The mother GUV lumen contained no fluorescent vesicles initially, but fluorescent small GUVs were observed after 58 s (e.g., 79 s and 112 s in Fig. 2A (2)). This fluorescence became apparent prior to the AF647 leakage, indicating that Rh-R_9_ enters the mother GUV lumen and binds to the small GUV membranes before the GUV membrane is damaged [19,20]. This experiment was repeated with 17 GUVs and all provided similar results. The entry of Rh-R_9_ into the GUV lumen was detected within 6 min for 10 GUVs, so the fraction of GUVs in which Rh-R_9_ entered before membrane damage among all examined GUVs (hereafter, the fraction of entry of Rh-R_9_) after 6 min interaction (i.e., *P*_entry_ (6 min)) was 0.59. AF647 leakage in these 10 GUVs occurred within 6 min, indicating that the fraction of leaking GUVs after 6 min interaction (*P*_leak_ (6 min)) was 0.59. We further examined the interaction of 5.0 μM Rh-R_9_ with GUVs without containing small GUVs in the lumen, and obtained a similar fraction of leaking GUVs, indicating that small GUVs do not affect membrane damage.

We sometimes observed the formation of fluorescent thin tubes in the mother GUV lumen that attached to the mother GUV membrane. Fig. 2B shows the interaction of 7.0 μM Rh-R_9_ with single GUVs. Several tubes were observed in the lumen between 13 and 114 s, then after 168 s, fluorescent small GUVs were observed in the mother GUV lumen. These tubes and small GUVs experienced rapid Brownian motion, and thus were not observed continuously because they could only be observed when they were located in the focal plane. The FI of the GUV lumen due to AF647 remained constant up to 6 min, indicating no leakage of AF647 and thus likely no membrane damage.

**Fig. 3:**
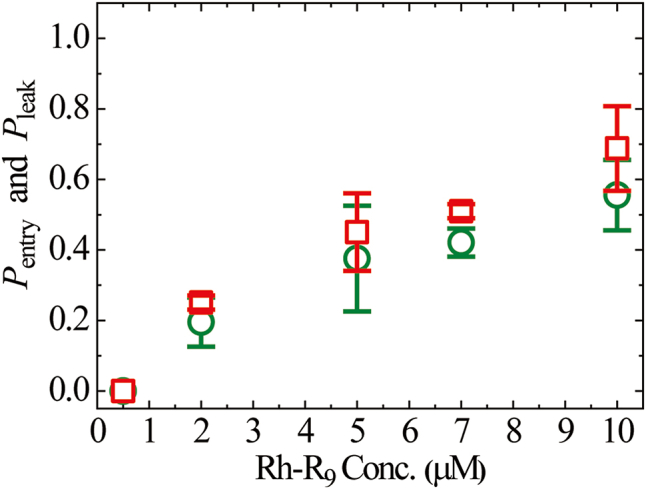
Rh-R_9_ concentration dependence of the fraction of leaking GUVs (*P*_leak_) and the fraction of entry (*P*_entry_). *P*_leak_ (6 min) (green ○) and *P*_entry_ (6 min) (red □). Mean values and SDs are shown. (For interpretation of the references to colour in this figure legend, the reader is referred to the Web version of this article.)

We examined the interaction of various concentrations of Rh-R_9_ with GUVs (*N* = 3−4). Most GUVs exhibiting AF647 leakage showed membrane damage after the entry of Rh-R_9_. We obtained the mean values and SDs of *P*_leak_ (6 min) and *P*_entry_ (6 min). Fig. 3 shows the peptide concentration dependence of *P*_leak_ (6 min): no leaking GUVs were observed at or below 0.5 μM, whereas at or above 2.0 μM *P*_leak_ (6 min) increased with peptide concentration, and at 10 μM *P*_leak_ (6 min) = 0.56. In contrast, for *P*_entry_ (6 min), no entry was observed at or below 0.5 μM, and at or above 2.0 μM *P*_entry_ (6 min) increased with peptide concentration, with *P*_entry_ (6 min) = 0.69 at 10 μM. These results indicate that the rate of Rh-R_9_-induced membrane damage and that of its entry into GUV lumen increase with peptide concentration. The peptide concentration dependence of *P*_leak_ (6 min) and *P*_entry_ (6 min) are very similar, supporting the above result that membrane damage occurs after the entry of Rh-R_9_. Tube formation in the mother GUVs was not related to the entry of Rh-R_9_ into the GUV lumen or to membrane damage.

## 4 General discussion

Rh-R_9_-induced calcein leakage from *E. coli* cells indicated that Rh-R_9_ causes cell membrane damage. After a large amount of calcein leaked, a significant amount of Rh-R_9_ entered the cytosol. This cell membrane damage occurs within 6 min after the interaction of Rh-R_9_ with cells starts, suggesting that the cell membrane damage is induced by the direct or indirect interaction of Rh-R_9_ with the cell membrane, not by cell death, because it takes a longer time to induce membrane damage after cell death. This result of Rh-R_9_ is different from that obtained using Rh-LfcinB (4–9) (i.e., significant entry of peptides into the cytosol without cell membrane damage) [17]. Recently, it was indicated that AMPs-induced damage of bacterial cell membrane causes their bactericidal activity at the single-cell level [28], and thus, the Rh-R_9_-induced cell membrane damage may be related to its antimicrobial activity. However, the binding of Rh-R_9_ with DNA and proteins in the cytosol may also contribute to its antimicrobial activity [17]. Since the lag time between the entry of Rh-R_9_ into the cytosol and membrane damage is ∼1 min, it is difficult to judge which factor plays more important role in its antimicrobial activity.

In contrast, Rh-R_9_ could enter the lumen of *E. coli*-lipid GUVs without damaging the membrane and then after its entry the GUV membrane was damaged. This result is different from that obtained using Rh-LfcinB (4–9) (i.e., peptides enter the GUV lumen, but no membrane damage occurs) [17]. The apparent different temporal correlation between fluorescent probe leakage and peptide entrance observed in *E. coli* cells and GUVs may be explained by a few causes. One is the different sensitivity of the detection of peptides in the cytosol and the GUV lumen: the single GUV method has higher sensitivity than the single cell experiments, because the binding of these peptides to the membranes of small GUVs in the mother GUV lumen greatly enhances their FI due to the low dielectric constant of the membranes and their condensation at the membranes [17,19,20]. In contrast, we detect the entry of Rh-R_9_ into cytosol of single cells from the FI of their cytosol, and the time of entry of the peptide is defined as the time when the FI of the whole cell rapidly increases. The other is the difference in the structure of cells and GUVs: *E. coli* cells have the outer membrane, peptidoglycan layers, membrane proteins in the cell membrane, and thus, the binding of R_9_ to these structures may retard its entry into their cytosol. However, irrespective of the apparent different temporal correlation, the membrane damage occurs in both cells and GUVs within several minutes after starting the interaction, suggesting that the mechanism of Rh-R_9_-induced membrane damage is similar for both cases.

The rate of Rh-R_9_-induced membrane damage judging from *P*_leak_ (6 min) in *E. coli* cells is higher than that in the GUVs, which may be explained by the presence of membrane potential in the cells [22].

The Rh-R_9_-induced membrane damage does not induce GUV burst, in contrast to AMP-induced burst of *E. coli*-lipid-GUVs [22,23] and dioleoyl-PE/DOPG-GUVs [29]. This interaction also contrasts with that of carboxyfluorescein-labeled R_9_ (CF-R_9_) with DOPG/DOPC-GUVs where no membrane damage occurs after its entry into the GUV lumen [24]. These results indicate that the lipid composition affects greatly the interaction of R_9_ with lipid bilayers.

## 5 Conclusions

Rh-R_9_ damages the membrane of *E. coli* cells immediately after the interaction between Rh-R_9_ and the cells begins. This damage allows a significant amount of Rh-R_9_ to enter their cytoplasm. In contrast, Rh-R_9_ enters the lumen of *E. coli* lipid-GUVs without causing AF647 leakage, but leakage does eventually occur, indicating that Rh-R_9_ damages the membrane of *E. coli* lipid-GUVs. These results indicate that Rh-R_9_ can damage the lipid bilayer region of a cell membrane, which may be related to its antimicrobial activity. Many studies to date have reported the penetration of CPPs into bacterial cells through a previously unknown mechanism [[13], [14], [15], [16]]. The present study clearly indicates that a significant amount of Rh-R_9_ enters the cytoplasm due to cell membrane damage.

## CRediT authorship contribution statement

**Sabrina Sharmin:** Writing – review & editing, Writing – original draft, Investigation, Formal analysis, Data curation, Conceptualization. **Md. Zahidul Islam:** Writing – review & editing, Methodology, Investigation, Data curation. **Masahito Yamazaki:** Writing – review & editing, Writing – original draft, Validation, Supervision, Software, Resources, Project administration, Investigation, Funding acquisition, Formal analysis, Data curation, Conceptualization.

## Declaration of competing interest

The authors declare that they have no known competing financial interests or personal relationships that could have appeared to influence the work reported in this paper.
